# Knowledge representation of motor activity of patients with Parkinson’s disease

**DOI:** 10.1007/s11047-014-9475-0

**Published:** 2014-12-31

**Authors:** Bożena Kostek, Adam Kupryjanow, Andrzej Czyżewski

**Affiliations:** 1 Audio Acoustics Lab, Gdansk University of Technology, Narutowicza 11/12, 80-233 Gdańsk, Poland; 2 Multimedia Systems Department, Faculty of Electronics, Telecommunications and Informatics, Gdansk University of Technology, Narutowicza 11/12, 80-233 Gdańsk, Poland

**Keywords:** Biomedical signal, Granular representation, Parkinson’s disease, ANN, SVM, Motor activity data processing

## Abstract

An approach to the knowledge representation extraction from biomedical signals analysis concerning motor activity of Parkinson disease patients is proposed in this paper. This is done utilizing accelerometers attached to their body as well as exploiting video image of their hand movements. Experiments are carried out employing artificial neural networks and support vector machine to the recognition of characteristic motor activity disorders in patients. Obtained results indicate that it is possible to interpret some selected patient’s body movements with a sufficiently high effectiveness.

## Introduction

Investigating biomedical signals may be treated as decomposing of knowledge into small entities of knowledge, called granules. Zadeh informal definition of granulation involves “decomposition of whole into parts” (Zadeh 1997). Pawlak pointed out that granularity of knowledge is inherently connected with the foundation of rough set theory. The concept of the rough set rely on classification of objects of interest into similarity classes, which form elementary building blocks (atoms, granules) of knowledge (Pawlak 1998). It should be stated that granular computing is now a well established field of research (Lin et al. 2002; Pedrycz et al. 2008; Polkowski and Skowron 1998; Wang 2007; Wang et al. 2009). Publications that may be found in the literature (Pedrycz et al. 2008; Wang et al. 2009) offer a comprehensive reference source for the granular computing community and also show that this area encompasses computational intelligence, fuzzy set theory, rough sets, etc. In books related to this domain one may found many case studies that involve capturing knowledge from sensors (Gacek and Pedrycz 2012; Pedrycz 2001). However, it should be emphasized that granular computing founds its way toward human-centric information processing, including biomedical signal analysis (Bargiela and Pedrycz 2009; Momot et al. 2010; Pedrycz and Gacek 2002). This paper presents classification of acceleration signals extracted from sensors placed on the Parkinson’s disease (PD) patient, decomposed into tri-axial entities.

PD belongs to the group of neurodegenerative diseases. It is a slowly progressing, degenerative disease of the central nervous system that is usually associated with disturbed dopamine production by the nervous cells of the brain. The disease manifests itself with movement disturbances. The cause of such disturbances has not yet been fully elucidated. The treatment of patients with PD is mainly based on minimizing the effects of symptoms. PD develops slowly and may last for many years. At its initial stage it is difficult to detect. It is crucial that once the disease is diagnosed, its fast development is restrained. Noticing changes in patient’s condition in a matter of short time is hard, especially that there are no fully objective tests that would allow for the assessment of its stage. The main symptoms of PD are associated with limitations in patient’s motor activity such as involuntary slowing of movements, bradykinesia, disturbances of gait including freezing of gait (FOG), disturbances of balance resulting in falls, problems with coordination of movements, resting effort tremor (Izworski et al. 2005; Kostek et al. 2012), difficulty swallowing, slowed mimical facial movements, etc. In addition to the symptoms associated with the motor activity, PD may also cause problems with concentration and planning of everyday activities.

One of the methods used for the assessment of patient with diagnosed PD is a series of standardized clinical tests performed by a specialist physician, called a *Unified Parkinson’s Disease Rating Scale* (UPDRS 2003). However, the results of these tests are encumbered with errors resulting from their subjective nature. The evaluation of patient’s condition with the UPDRS tests conducted every 3 or 6 months allows for the assessment of PD’s progression, but they require regular appointments with the attending physician, which is not always possible. In such cases, it may not always be achievable to verify the exact time when the medicines were taken or examine the side effects of the drugs which usually manifest themselves as dyskinesias (involuntary movement disorder) (White et al. 2006). With time, the effectiveness of the treatment begins to decrease gradually and the so-called “on” and “off” phases occur in an alternating mode (periods of good motor ability and significantly worse motor ability, respectively). The changes in the physical condition are clearly associated with the rhythm of drug taking and, in the case of the “on” phase, can be predicted. However, some patients experience sudden “off” phases without any obvious connection with medicine intake. We may also observe a phenomenon called an “on–off” phase, which involves multiple and quick switches between mobility and immobility. Therefore, too rare and irregular follow-up visits at the doctor’s office lack to provide information of a possible deterioration of the patient’s condition. Also, they do not enable to establish whether a given medicine or its dosage is appropriate and, most importantly, whether the patients reporting to a doctor is currently in the “on” or “off” phase (Zwan et al. 2010). This makes it impossible for a physician to fully assess the condition of a PD patient.

In order to support the process of the evaluation of patients’ condition, there are some systems created for constant monitoring. The system created within the PERFORM project with the participation of the authors of this paper can serve as an example of that approach (*“A soPhisticatEd multi*-*paRametric system FOR the continuous effective assessment and Monitoring of motor status in Parkinson’s disease (PD) and other neurodegenerative diseases progression and optimizing patients’ quality of life”*) (Baga et al. 2009; Greenlaw et al. 2009; Kupryjanow et al. 2010; Maziewski et al. 2009). The system is supposed to monitor the patient’s condition 24-h a day on the basis of biomedical signals analysis. It uses specially-designed sensors located on the patient’s body and a series of tests conducted on diagnostic appliances (Baga et al. 2009; Greenlaw et al. 2009). The patient is monitored while being at home and the information obtained after the initial processing of the collected signals is sent to the hospital central unit. The unit then performs a detailed analysis of the data received. This may enable the assessment of the correctness and effectiveness of individually matched schemes of treatment and their possible adaptations. The monitoring of patients with PD mainly involves information obtained from acceleration sensors (accelerometers), gyroscopes, electrooculogram, spirometer and sensors of pressure with an analysis of the video footage recorded during the tests mentioned earlier in the text. Also, monitoring PD patients by employing data from patients’ diaries which were rated by clinicians in the UPDRS scale and rule-based data processing was proposed in which metadata on patients were processed using rough sets (Zwan et al. 2010).

The issue of movement categories recognition on the basis of acceleration signals analysis was investigated in numerous studies (Bao and Intille 2004; Godfrey et al. 2008; Lee and Mase 2002; Lombriser et al. 2007; Mathie et al. 2004). However, the classification of movement categories in patients with PD constitutes an individual problem, since it requires taking into account the disruptions resulting from movement disturbances of the patients (Tadeusiewicz 2009; White et al. 2006). The algorithms responsible, for example, for the analysis of hand movement may erroneously interpret intense shaking as an intended limb movement. Gait recognition is limited by a fact that PD patients frequently perform movement similar to walking when they are at rest, thus recognizing walking/no-walking and hand movement/no-hand movement and the nature of these movements are important for the assessment of the patients or the diagnosis of the PD.

This article presents the algorithms enabling to classify selected movement categories in patients with PD. They were designed to support the evaluation of PD progression. Even though the algorithms utilized are conventional, i.e. SVM and ANN, they results in an effective recognition of the following categories: walking/no walking, hand movement/no hand movement. The classification is carried out on the basis of the analysis of acceleration signals coming from tri-axial accelerometers located on the patient’s body. The last part of this paper discusses another study, which show the possibility of hand motor activity analysis in PD patients conducted by processing the image recorded by an Internet video camera on a stand. This device is called Virtual TouchPad (VTP). Hand movement tests are included in the UPDRS examination. Automatic performance of such tests is an alternative to testing hand motor function with the use of gloves containing, for example, grip sensors or accelerometers (Kostek et al. 2012; Lee and Mase 2002).

## Recording biomedical signals

For PD patients’ monitoring a multimedia application was conceived and engineered. In order to test and train the classifiers recognizing the categories of movement activity in patients with PD, we used acceleration signals recorded during the tests involving patients and doctors from the Neurology Department of the St. Adalbert’s Hospital in Gdansk and the hospital in Ioannina (Greece). The experiment included 33 patients (mean age 68.2 years; standard deviation 9.8 years), average illness duration time was 9 (SD ± 5) years including both men and women in approximately the same number. The patient was supposed to perform a series of movements simulating everyday activities. The sequences of the performed movements and the recording were carried out in controlled conditions to make it possible to ascribe particular labels of movements to the signals at the stage of initial processing. Since PD is an asymmetrical disease, most of these symptoms were assessed separately for both right and left body sides.

Each of the patients performed the following activities: walking in straight line with a turn, lifting an object with the left, right and both hands, lifting the left, right and both hands, sitting and getting up from a chair, lying down and getting up from bed, standing, sitting and lying. Each of the above was performed in a sequence of dynamic and static activity. For example, gait sequence was recorded in the following sequence: standing in place, walking with a turn and stopping. Each sequence of movement was repeated three times. During the recording of acceleration signals we also recorded the video image synchronized with the acceleration signals. Video records allowed for precise tagging of the beginning and end of each of movement categories.

**Fig. 1:**
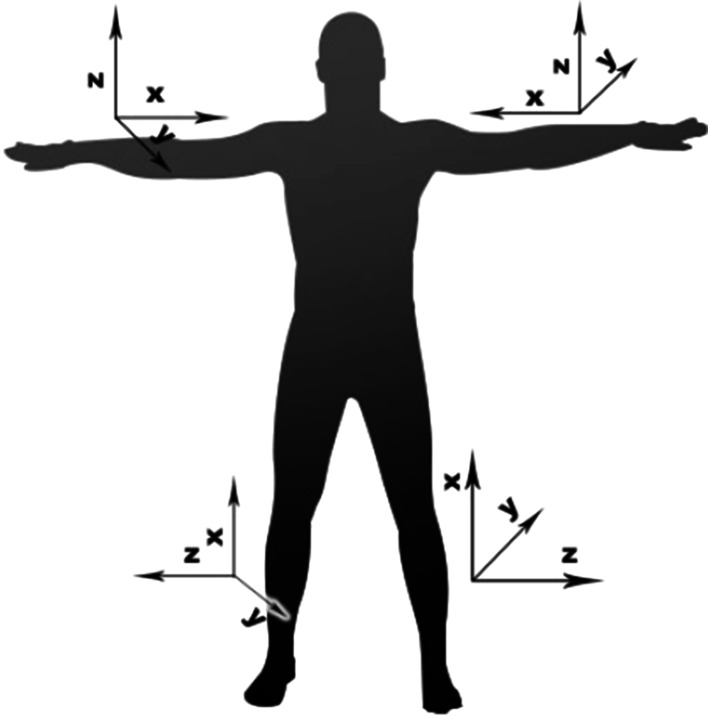
Location of acceleration sensors on subject’s body

**Fig. 2:**
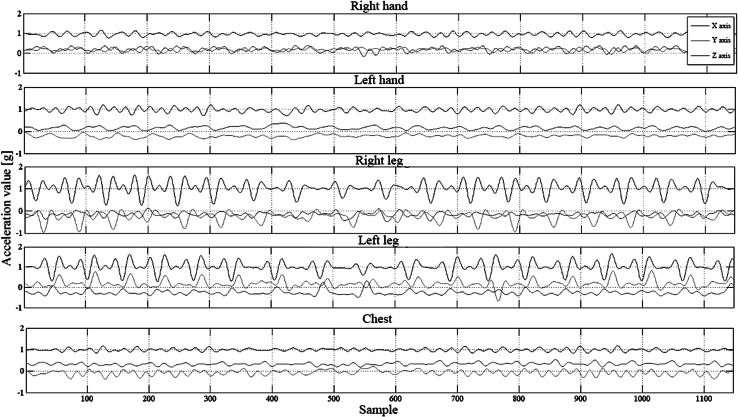
Acceleration signals recorded during the tests

Acceleration signal were recorded with two types of devices equipped with triaxial accelerometers. The signal was recorded on *microSD* cards integrated with the recording devices. During the tests we used the devices produced by *Shimmer* (Shimmer 2008) and the device designed within the *PERFORM* project system (Baga et al. 2009; Greenlaw et al. 2009). The accelerometers were located on subjects’ wrists, ankles and chests. This was for the purpose to detect the highest number of movement activities and assess patient’s condition automatically by the algorithms recognizing the symptoms of the disease. The number of sensors was limited so that not to cause any discomfort to the patient when using the device. The range of accelerations recorded by the accelerometers fell within the range of ±6 g, which fully covers the range of accelerations obtained during typical movement activities of a human body. The sampling rate of the signal was 51.2 Hz for *Shimmer* and 62.5 Hz for the *PERFORM* system. The location of the devices on subject’s body is presented in Fig. 1. In the presented study signals from 5 accelerometers and 3 axes were independently recorded, resulting in 15 acceleration time-domain signals. Examples of acceleration signal are presented in Fig. 2.

On signal diagrams pertaining to the *x* axis, constant component equal to the gravity acceleration can be observed (1 g). The presence of the constant component for this axis is associated with the orientation of the accelerometers, which had been located in the following way: axis *x*—vertically, *y*—horizontally and perpendicular to the chest, axis *z*—vertically and parallel to the chest.

## The parameterization of acceleration signals

The analysis of acceleration signals is usually carried out after signal parameterization is performed (Huynh and Schiele 2005). In the case of the classification of signals recorded during tests with PD patients it is necessary to additionally pre-process the signals in order to eliminate the interferences resulting from the symptoms of the disease. Earlier studies conducted in the Multimedia Systems Department (MSD), Gdansk University of Technology, GUT (Kupryjanow et al. 2010; Maziewski et al. 2009) allowed a conclusion that low-pass acceleration signal filtering with the cut-off frequency of 3 Hz gives the best results in interference elimination, at the same time preserving significant information associated with the frequency of movement. One may also find that the high-pass cut-off frequency of 2 Hz is used for acceleration signals (Rissanen et al. 2011). Therefore, all signals had been exposed to a low-pass filtering with the use of a IIR filter of a 3 Hz cut-off frequency.

The parameterization of the signals was carried out in time windows dependent on the type of activity. In the case of gait recognition, a window size of 1,250 ms was used with hop size of the 625 ms, which corresponded to 64 and 32 signal samples overlapping between consecutive frames for *Shimmer* and 78 and 39 samples for the devices of the *PERFORM* system. For the parameterization of the signal used for hand movement classification we used a shorter window of 625 ms and the hop size of 320 ms. This was associated with the necessity to detect high-frequency activities. All the parameters were normalized to the value of <−1,1> before being fed to the input. As mentioned before, acceleration signals extracted from sensors form tri-axial entities which may be treated as sources of granules of information. Especially important seems a granular interpretation of their nature in the context of a human-centric description of relationships existing within data (Gacek 2013). Moreover, because of much redundancy contained in the acceleration signals, there is a need to parameterize these signal entities. Thus if we recall that the essence of granularity (Pedrycz 2001) is a meaningful representation of a collection of numeric values (real numbers), i.e. {*x*_1_, *x*_2_, …, *x*_*N*_}, then the extracted feature vectors will become information granules. The parameters describing information extracted from the acceleration signal frames can be divided into the ones calculated in the time domain and the spectrum domain. They will be discussed in Sects. 3.1 and 3.2, respectively.

### Parameters in time domain

For the parameterization, we first used the standard statistical signal metrics. The first one was the mean value of the signal—a parameter describing the level of acceleration for a given frame of signal. It can be observed that this parameter is of high values for dynamic activities (e.g. walking, hand movement) and low for static activities (e.g. sitting). The mean value of the acceleration signal can be counted in the following way:

1 $$ \overline{x} = \frac{1}{N}\sum\limits_{n = 1}^{N} {x(n)} $$

where *n* is the number of acceleration signal sample, and *N* is the length of a signal frame in samples.

Standard deviation represents the range of signal variability:

2 $$ std(x(n)) = \sqrt {\left( {\frac{1}{N - 1}\sum\limits_{n = 1}^{N} {\left( {x(n) - \overline{x} } \right)^{2} } } \right)} $$

Another parameter—kurtosis was determined in order to establish the dynamics of changes in acceleration signal:

3 $$ krt(x(n)) = \frac{{m_{4} (x(n))}}{{std(x(n))^{2} }} - 3 $$

where *m*_*4*_(*x*(*n*)) represents the fourth central moment.

The crest factor is a ratio of the maximum signal value to the RMS value. It describes the impulsiveness of the signal:

4 $$ k_{sz} (x(n)) = \frac{\hbox{max} (x(n))}{{\sqrt {\frac{1}{N}\sum\limits_{n = 1}^{N} {x(n)^{2} } } }} $$

In order to describe the relation between the movement of particular limbs and the body, a correlation coefficient was calculated corresponding to the same axes of accelerometers located on different body parts. As a result, 30 coefficients were obtained for five sensors. Additionally, a correlation coefficient was determined between each pair of axes of the same accelerometer, which completed the description of the movement by the relation of a temporary location of the sensor. The correlation coefficient was calculated using the following formula:

5 $$ corr(x(n)_{i}^{l} ,x(n)_{j}^{m} ) = \frac{{\overline{{x(n)_{i}^{l} x(n)_{j}^{m} }} - \overline{{x_{i}^{l} x_{j}^{m} }} }}{{std(x(n)_{i}^{l} )std(x(n)_{j}^{m} )}} $$

where *i* = 1…5, *j* = 1…5 represent the numbers of acceleration sensors and *l* = {*x, y, z*}, *m* = {*x, y, z*} are the axes of accelerometers. The correlation coefficient between the sensors was calculated for *i* ≠ *j* and *l* = *m,* correlation coefficients between axes of accelerometers for *i* = *j* and *l* ≠ *m*.

### Energy-based descriptors

The complexity of movement was described with the use of the signal spectrum energy expressed in the following formula:

6 $$ E(A(k)) = \frac{{\sum\limits_{k = 1}^{K} {A(k)^{2} } }}{K} $$

where *A*(*k*) is *k*-*th* amplitude spectrum line of the acceleration signal, *K* denotes the total number of spectrum lines.

The evaluation of periodicity is described by the entropy:

7 $$ Ent = - \sum\limits_{k = 1}^{K} {p(k)\log_{2} \left( {p(k)} \right)} $$

where *p*(*k*) represents the probability of *A*(*k*) value occurrence in the amplitude spectrum of the acceleration signal. Low value of entropy indicates the periodicity of the analyzed signal.

## Classifiers

The classification of acceleration signal was carried out using two independent classifiers designed for the recognition of the gait category and hand movement. The classifiers involved artificial neural networks (ANN) and the support vector machine (SVM) (Vapnik 1995). Due to the fact that classifiers based on ANNs are widely-used in many domains, the description of algorithm settings and parameters will be limited to the most important information concerning the structure and parameters of classifiers. On the other hand, some basic assumptions of the SVM algorithm will be given. In order to find an optimal placement of the accelerometers (i.e. a placement which allows for the classification of a given movement category at the highest possible effectiveness), we prepared classifiers allowing for a recognition of movement activities with the use of a various number of sensors. Also, in the literature one may find investigations to determine the optimal placement of accelerometers for the purpose of detecting a range of everyday activities (Cleland et al. 2013).

### SVM algorithm

The method was originally invented by Vapnik (1995), as a classifier allowing for dividing sets of parameters into two classes **y** = {*y*_*1*_ *, y*_*2*_ *, …, y*_*d*_}, where *y*є{−1,1}. The classification is based on the assumption that it is possible to separate the sets of parameter vectors **x** = {*x*_*1*_ *, x*_*2*_ *,…, x*_*d*_} belonging to the *R* ^*n*^ space into two subsets *U*_*1*_ and *U*_*2*_, using a linear function *f*(**x**) expressed by the following formula (8):

8 $$ f({\mathbf{x}}) = {\mathbf{w}} \cdot {\mathbf{x}} + b $$

A set of all parameters {**x, y}** є *U.* Plane *f*(**x**) separating the set of parameters is called a hyperplane. The training of the classifier involves finding a hyperplane separating two parameter sets with preserving of the largest possible margin, where the established hyperplane is counted as *f*(**x**) = 0, and the margin as *f*(**x**) = −1 and *f*(**x**) = +1. The highest value of the margin is achieved if the value of (9) is minimal and the assumptions described in formulas (10) and (11) are fulfilled.

9 $$ \hbox{min} \frac{1}{2}\left\| {\mathbf{w}} \right\|^{2}\,+\,C\sum\limits_{i}^{l} {\zeta_{i} } $$

10 $$ y_{i} \cdot \left( {\left( {{\mathbf{w}} \cdot x_{i} } \right) + b} \right) \ge 1 - \zeta_{i},\,\forall_{i} \in U $$

11 $$ \zeta_{i} \ge 0,\forall_{i} \in U $$

where ||**w**|| is the length of the vector **w**, *C* is a cost, and *ζ*_*i*_ is a slack variable determined independently for each vector *x*_*i*_. Including the cost and the slack variable allows for dividing the nonlinearly separable sets. The value of the cost is determined by the user during the training of the classifier. The higher the value of the parameter *C*, the lower the value of the margin, which may result in excessive matching of the classifier to the training data.

Primarily the SVM method did not take into consideration the case of nonlinearly separable sets. Developing the method by adding the non-linear division of parameters required an introduction of the kernel function, which allows for the transformation of **x** parameters from the *R* ^*n*^ space to the *R* ^*m*^, space, where *m* > *n*. It is presupposed that after the transformation to the space of a higher dimension, i.e. the feature space, the sets will be separable and it will be possible to separate them using a linear function. Once the *Φ,* function mapping the parameters to the feature space has been taken into account, the function describing the hyperplane will take on the following form (12):

12 $$ f({\mathbf{x}}) = {\mathbf{w}} \cdot \varphi ({\mathbf{x}}) + b $$

where *φ*(***x***) represents the mapping function. Just as in the case of separable data using a linear function, for the training of non-linear classifier the highest value of the margin between the hyperplanes *f*(**x**) = −1 and *f*(**x**) = +1 is determined. Once the mapping function has been included, the conditions which allow for the highest value of the margin can be determined in the following formula (13), taking into account the limiting conditions (14) and (15).

13 $$ \hbox{min} \frac{1}{2}\left\| {\mathbf{w}} \right\|^{2}\,+\,C\sum\limits_{i}^{l} {\zeta_{i} } $$

14 $$ y_{i} \cdot \left( {\left( {{\mathbf{w}} \cdot \varphi (x_{i} )} \right) + b} \right) \ge 1 - \zeta_{i},\,\forall_{i} \in U $$

15 $$ \zeta_{i} \ge 0,\,\forall_{i} \in U $$

The mapping function *φ*(***x***) introduces an additional complexity into the calculations. In order to avoid the necessity of knowing the mapping function the so-called kernel trick can be used, which is described by the following formula (16):

16 $$ K(x,z) = \varphi (x) \cdot \varphi (z) $$

where *K*(*x, z*) denotes the kernel function, and *φ*(***x***) and *φ*(***z***) the mapping functions. Thanks to relation (16) it is possible to omit the mapping functions during the training of the classifier and replacing them with the kernel function. This allows for a simplification of calculations conducted during the training.

### Walk recognition classification

Gait classifier is to distinguish particular fragments of a signal in which the subject is walking from fragment in which he/she is performing any other activities (e.g. lying down in bed, sitting on a chair). Decisions made by this classifier can be used by the algorithms assessing, for example, FOG (UPDRS14—“Freezing when walking”). Since it was necessary to include the possibility of gait recognition (UPDRS29—“Gait”) with the use of a various number of sensors, a various number of parameters was fed to the input. Table 1 presents the relation between the number of parameters and the number of sensors used in the analysis.

**Table 1:** The relation between the number of sensors and the number of parameters

Number of sensors	1	2	3	5
Number of parameters	21	45	72	135

The neural network used had one hidden layer. The number of neurons in the input layer was dependent on the number of accelerometers used for the classification (see Table 1). The number of neurons in the hidden layer was calculated according to the following formula:

17 $$ n_{ukr} = \frac{{n_{wej} }}{2} + n_{wyj} $$

where *n*_*in*_ is the number of neurons in the input layer, and *n*_*out*_ in the number of neurons in the output layer.

The ANN-decision-based system for gait classification had two output neurons. If the data belonged to the ‘gait’ class, the value at the output was expected to be [1,0]. Otherwise value [0,1] was expected. The value of ‘1’ was ascribed to the output which returned the highest value. For the training network we used error back propagation algorithm. Neurons of both network layers used the sigmoid function of activation defined in the following formula:

18 $$ f(x) = \beta \frac{{(1 - e^{\alpha x} )}}{{(1 + e^{\alpha x} )}} $$

where *α* and *β* were equal to a unit.

The structure of networks used for the classification of hand movement and gait consisted of the input, one hidden and the output layers. The input layer was dependent on the number of the sensors used

The classifier based on the SVM was created using the C-support vector Classification algorithm (Boser et al. 1992; Chang and Lin 2010). The Gaussian kernel function was used calculated with the following formula:

19 $$ K(x_{i} ,x_{j} ) = e^{{ - \frac{{||x_{i} - x_{j} ||}}{{2\gamma^{2} }}}} $$

For the training of the classifier based on the SVM method two different approaches to (*C*) and *γ* cost parameters selection were applied. Initially, we established fixed values of parameters *C* = 62.5 and *γ* = 0.5, regardless of the number of acceleration sensors that were used. The values of parameters were selected experimentally. In the second attempt, in order to find the parameters ensuring the best effectiveness of classification, we used the grid-search method available in the OpenCV library. This method is based on dividing the area of searching with a conventional net and then using for data calculations two parameters which change in every iteration. Finally, a pair of parameters *C* and γ, is chosen, which provides the best accuracy of recognition (Weka system). It should be pointed out that it is extremely crucial to find such a pair of parameters *C* and γ, so that the classifier recognizes unknown data in the most efficient way. At the second attempt values of parameters *C* and *γ* were obtained depending on the number of accelerometers that were used.

### Hand movement recognition

Hand movement classifier was designed to recognize the activity of one or two hands being moved. Information concerning the currently performed activity may be used for example in the process of bradykinesia assessment. The classifier analyzed only those fragments of the signal, which had not been marked by the gait detector as gait.

The neural network was in this case of almost the same structure as in the case of gait classification. The difference between the structures was in the number of network output. Four output neurons were used, each corresponding to the possible classes of hand movement: left, right, both hands, no movement. The training process was conducted the same way as in the case of gait classification. Also, the same algorithm of training and activation function were used as in the detection of gait.

Since the classifier based on the SVM is by definition a two-class classifier, thus in the case of hand activity detection it required more sophisticated scheme with a possibility of classification of numerous body movement classes. In this case the *one*-*versus*-*all* method was applied. The method requires creating a number of two-class classifiers (the number of classifiers is equal to the one of the classes), all of which are supposed to distinguish one of the classes from the remaining ones. During the recognition of movement, the vector of the parameters is fed to the input of numerous classifiers, and the final decision as regards the assigning to a particular class is made on the basis of the certainty of the decision made by a classifier. If several classifiers make a decision that a vector belongs to a class recognized by them, finally the class for which the decision of the highest estimated probability has been made will be selected. The probability of decision making is calculated on the basis of the SVM model and the necessary implementation is available within the libSVM library (Chang and Lin 2010).

## Experiments and results

Below are the results showing the effectiveness of movement activity recognition according to the algorithm of classification used and the testing method. Each algorithm was testes with the use of the cross-validation algorithm and the leave-one-out method (in this case the *N*-element sample is divided into *N* subsets, containing one element each). In the leave-one-out method the classifiers were trained in the following way: the training set contained parameters corresponding to movement activities performed by *n*−1 people, where *n* is the number of all patients, while the testing set contained the parameters for one person. The values of effectiveness presented in Tables 2 and 3 are the mean values for all 33 patients. Since the testing phase is conducted with the use of parameters extracted from patients’ body motion that were not used for training the classifier, the leave-one-out method allows for the evaluation of the generalization capabilities of algorithms recognizing motor activities.

**Table 2:** Results of gait recognition—*leave-one-out* method

Accelerometers’ configuration	Type of activity	SVM (*C* = 62.5, *γ* = 0.5)		SVM grid-search		ANN	
		Effectiveness	SD	Effectiveness	SD	Effectiveness	SD
1—left leg	Gait	95.63	7.34	96.34	**5.89**	**97.27**	17.82
	No gait	97.66	7.76	**97.86**	**7.57**	97.00	7.90
1—right leg	Gait	**95.06**	**17.65**	94.93	18.69	94.86	17.97
	No gait	96.96	8.82	**97.18**	8.87	96.04	**8.06**
2—legs	Gait	96.71	13.27	97.51	10.91	**98.00**	**5.73**
	No gait	**98.86**	**3.16**	98.62	4.23	96.82	7.28
2—right leg, chest	Gait	97.15	9.71	**98.24**	**6.97**	96.35	14.00
	No gait	96.67	9.48	**97.75**	**7.08**	95.88	9.73
3—legs, chest	Gait	98.82	2.03	**99.19**	**1.66**	85.49	20.82
	No gait	**98.12**	6.08	98.01	**6.04**	77.10	16.77
3—left hand, right leg, chest	Gait	97.02	6.74	**98.01**	**4.96**	91.02	13.63
	No gait	**97.33**	**6.51**	97.31	6.95	86.28	17.81
5—legs, hands, chest	Gait	96.94	6.86	**97.16**	**6.65**	34.35	28.99
	No gait	**99.24**	**1.91**	98.36	3.71	83.89	19.60

**Table 3:** Results of gait recognition—cross-validation method

Accelerometers’ configuration	Type of activity	SVM (*C* = 62.5, *γ* = 0.5)		SVM grid-search		ANN	
		Effectiveness	SD	Effectiveness	SD	Effectiveness	SD
1—left leg	Gait	**97.47**	1.14	97.03	**0.97**	97.42	1.39
	No gait	**98.39**	0.88	98.31	0.86	98.30	**0.76**
1—right leg	Gait	98.37	0.89	**98.65**	**0.51**	97.50	1.30
	No gait	**99.09**	**0.54**	99.01	0.71	98.58	0.76
2—legs	Gait	**98.89**	**0.44**	98.99	0.72	98.68	0.90
	No gait	**99.53**	**0.41**	99.50	0.46	98.55	0.79
2—right leg, chest	Gait	98.62	1.07	**99.14**	**0.46**	98.66	0.67
	No gait	**99.26**	**0.42**	99.16	0.53	97.77	1.41
3—legs, chest	Gait	99.37	**0.37**	**99.69**	0.42	90.54	8.01
	No gait	**99.78**	**0.26**	99.69	0.29	89.10	12.95
3—left hand, right leg, chest	Gait	98.49	1.65	**98.91**	**1.19**	92.64	8.13
	No gait	**99.36**	0.34	99.35	**0.31**	85.04	19.11
5—legs, hands, chest	Gait	98.38	2.35	**98.99**	**1.29**	38.51	16.23
	No gait	99.65	0.37	**99.69**	**0.27**	94.04	7.48

### Walk recognition results

Tables 2 and 3 present the effectiveness of gait classification according to the number of accelerometers used and the type of the classifier. Apart from the effectiveness, we also counted the standard deviation in order to show the differences in the results obtained for particular persons (the leave-one-out test) and successive validations (cross-validation).

The result of the highest effectiveness and the lowest standard deviation for a given configuration of accelerometers was presented in bold. The best results of classification obtained from a particular testing method were highlighted by an underline. Based on the analysis of testing with the leave-one-out method (Table 2), it can be observed that the highest level of detection effectiveness was achieved for the SVM classifier. The best effectiveness for the neural network were achieved in only two situations (accelerometer on the left leg—gait recognition; accelerometer on both legs, category—no gait). The SVM classifier provided the highest global effectiveness of recognition. The most precise gait recognition was achieved when three accelerometers were used (legs, chest), while the opposing class was best recognized when all the available sensors were used. The results collected during algorithm testing with the cross-validation method (Table 3) in the case of gait detection correspond to the results from Table 2 (the highest effectiveness was obtained from the SVM classifier in the leg-chest configuration of accelerometers). The same configuration of accelerometers also enabled the most effective classification of the opposing class.

The analysis of the effectiveness of gait category recognition according to a method, accelerometers’ configuration and type of a classifier showed that the highest effectiveness (with the lowest possible number of sensors) can be obtained with the use of the SVM classifier and analyzing the signals recorded by three accelerometers (legs, chest).

### Hand movement recognition results

Tables 4 and 5 present the results obtained during algorithm tests of hand movement recognition. Apart from the effectiveness of classification and standard deviation, we also used the false-negative error values, which is a number (percentage) of erroneously classified examples of a given class. The tables contain the values pertaining to globally the best effectiveness and the best efficiency obtained for a given configuration of accelerometers.

**Table 4:** Results of hand movement recognition—*leave-one-out* method

Accelerometers’ configuration	Type of activity	SVM (*C* = 60, *γ* = 0.5)			ANN		
		Effectiveness	SD	2nd order error	Effectiveness	SD	2nd order error
2 sensors—subject’s wrists	Left	76.33	35.90	**1.43**	**81.56**	**33.55**	4.46
	Right	74.88	**35.31**	**0.44**	**77.85**	38.02	1.54
	Both	**93.15**	**13.15**	**4.30**	90.89	18.69	5.36
	No mov.	99.41	0.91	50.06	**99.63**	**0.84**	**38.70**
3 sensors—subject’s wrists, chest	Left	70.41	35.74	**1.76**	**77.22**	**33.03**	6.68
	Right	71.50	**37.75**	**0.36**	**74.95**	39.96	2.37
	Both	**90.40**	**15.96**	**4.64**	86.21	23.15	7.30
	No mov.	**99.69**	**0.60**	61.23	98.92	3.15	**46.35**

**Table 5:** Results of hand movement recognition—cross-validation method

Accelerometers’ configuration	Type of activity	SVM (*C* = 60, *γ* = 0.5)			ANN		
		Effectiveness	SD	2nd order error	Effectiveness	SD	2nd order error
2 sensors—subject’s wrists	Left	**92.12**	**3.34**	3.94	81.81	4.10	**3.47**
	Right	**89.93**	**2.88**	2.64	70.62	5.19	**1.04**
	Both	**93.60**	**2.30**	**0.95**	81.00	4.44	3.80
	No mov.	**99.89**	**0.05**	**16.94**	99.69	0.13	58.57
3 sensors—subject’s wrists, chest	Left	**88.52**	**3.22**	**2.94**	82.12	4.92	3.46
	Right	**90.12**	3.56	3.27	75.70	**2.21**	**1.35**
	Both	**96.51**	**1.64**	**0.99**	87.53	2.49	8.01
	No mov.	**99.96**	**0.04**	**17.72**	99.74	0.11	42.11

Based on a comparison of results it can be observed that in the case of cross-validation (Table 5) the best effectiveness of movement category recognition was obtained for the SVM classifier. However, the test conducted with the *leave-one-out* method shows that it is the neural network that has the best capability to generalize (in almost all cases shows the highest effectiveness).

As the data in Tables 4 and 5 show, the best results of hand movement classification were obtained when using the neural network and analyzing only the signals recorded by the sensors on subject’s wrists.

## Virtual TouchPad

This section describes a device called a VTP, which is based on hand image analysis. It is still being tested and has not yet been used in clinical studies.

Recently, more and more study groups have been working on finding possible solutions that would allow for automatic assessment of hand motor activity in patients with PD (Okuno et al. 2006; Shima et al. 2008). Hand motor activity is assessed by specialist physicians within the UPDRS tests (pertains to UPDRS23—“Finger tapping test”, UPDRS24—“Hand movements test”, UPDRS25—“Alternating hand movements test”). Automation of these tests allows the patient to perform them on his/her own at home and send the results to a specialist, who can analyze them at any moment. The assessment of hand activity includes the UPDRS mentioned above.

**Fig. 3:**
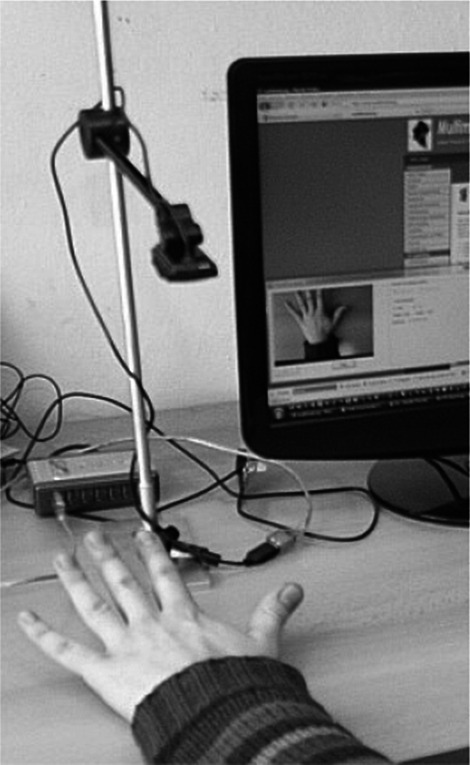
Mounting the camera on a stand

Most of the proposed solutions are based on technologies that require different types of sensors (e.g. accelerometers or proximity sensors) to be fastened to patient’s fingers or palm. Such systems are not comfortable and their use may cause problems for patients with advanced PD. Thus, methods that do not involve sensors are used as well. They are predominantly based on the analysis of hand image recorded by a video camera during test performance. Such a solution was used in the study by the MSD, GUT. The hardware layer of the system includes a computer and an Internet video camera on a specially-designed stand. The camera must be located in such a way that it can record the image of a hand placed parallel do the surface of a table/desk. Figure 3 presents a photo showing how to locate the camera on a stand. Specially designed algorithms of image processing allow for the detection of gestures made by a patient. The analysis of gestures then allows for presenting the result in the form of a diagram showing time taken by each gesture.

### Image processing with VTP

**Fig. 4:**
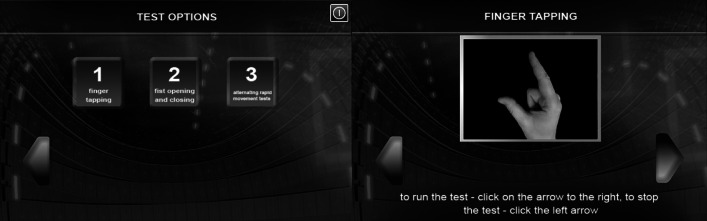
Application for hand movement tests

The algorithms of image processing were implemented with the use of the OpenCV library. The processing of the image involves seizing the picture form the video camera, recognizing hand gestures and producing results describing the movement. Image processing consists of two main steps: hand detection from the image and gesture recognition. The tests are conducted with the use of a specially-designed application which prompts how a given test should be performed and shows the results. Figure 4 presents sample screenshots from the application designed for hand tests.

#### Hand detection in VTP

Hand detection is based on the algorithms of background removal and contour detection based on the captured video. The algorithm of background removal is based on a static model of a scene, which is built at the so-called algorithm initialization stage requiring 50 video frames. The background model is a set of two values representing each of the image pixels, i.e. the mean pixel value designated from the initializing images and the averaged differences between the same pixels in successive frames. A given pixel is considered a foreground object, if the value of its intensity exceeds the range of the defined model. Once the background is removed, we obtain a binary mask representing the foreground. The unusual location of a video camera results in a shadow, which adds to the image with removed background. This makes hand detection more difficult. Thus, it is then necessary to apply the algorithm of shadow removal. The implementation involved using a typical algorithm based on image thresholding in YCrCb colour space, where the comparison is conducted by analyzing the background pixels and objects from the foreground (Prati et al.). Once the shadow is removed, a morphological operation of closing is carried out on the image mask, thanks to which any defects are eliminated from the image. Finally, the algorithm of contour recognition designates a hand contour. When the picture is being analyzed, it is presupposed that the image of the hand should have a surface of at least 5000 pixels.

#### Gesture recognition in VTP

**Fig. 5:**
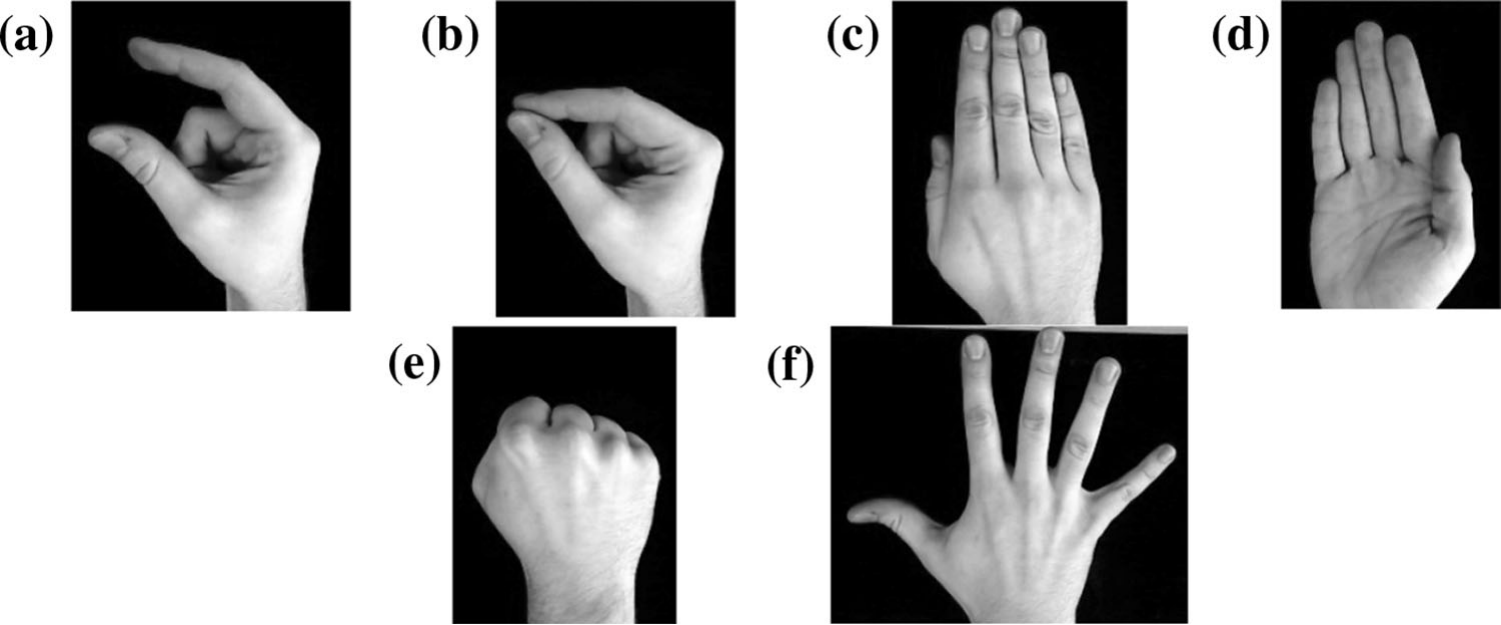
Gestures recognized by the classifiers: (**a**, **b**) finger tapping test (UPDRS 23), (**c**, **d**) opening fist/closing fist (UPDRS 24), (**e**, **f**) pronation–supination hand movements (UPDRS 25)

The algorithm of gesture recognition analyses the image mask which contains a designated hand contour. Gesture classification involves the SVM method. Just as in the case of hand movement analysis described in Sect. 4.2, for the detection of a number of gesture classes we used the *one*-*versus*-*all* method. Three independent classifiers were established. Each of them was supposed to recognize different gestures depending on the type of UPDRS test in which it was used. The designed classifiers distinguished the following types of gestures: adjoining fingers/separated fingers (Fig. 5a, b) in the finger tapping test (UPDRS 23), opening fist/closing fist (Fig. 5c, d) in the hand movement test (UPDRS 24), pronation–supination movements of hands (Fig. 5e, f) in the alternating hand movements test (UPDRS 25).

The mask of a hand contour was exposed to parameterization that involved finding the number of points in the image that contain the 1 value. The pixels were counted at the section from the centre of the image mask to the edge. 180 sections were defined and each successive one was turned from the previous one by 2°. As a result, 180 parameters were obtained describing each image mask.

The training of the SVM classifier was conducted independently for each of the users. 35 frames containing a given gesture were used to train each gesture.

### Assessment of hand motor activity using VTP

**Fig. 6:**
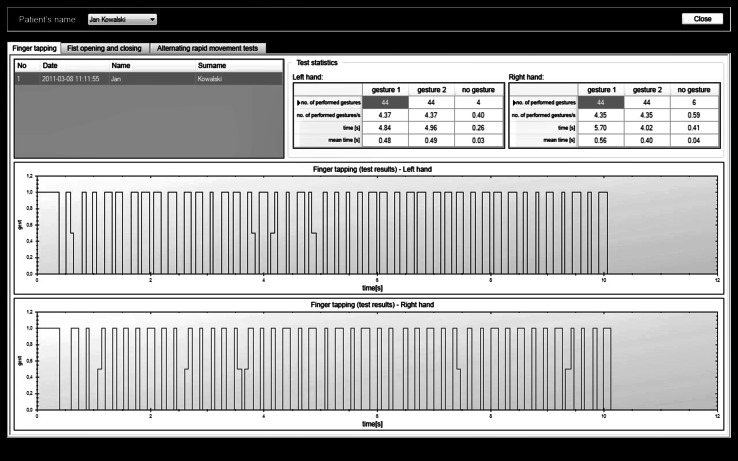
Example of results obtained

The hand motor activity test was carried out with the use of the application described in Sect. 6.1. It allowed for conducting three independent UPDRS tests. Each of the tests was performed independently for left and right hand. Figure 6 presents a screenshot from a program with the UPDRS 23 test results.

In this case, it can be observed, that the test was performed correctly (no problems with performing the activity), since at this stage of the study only healthy subjects with no disturbances of motor function were tested. The application does not provide assessment in the UPDRS scale but records objective parameters allowing the doctor to interpret them. The result of each of the tests was presented in a form of a diagram showing the interrelation between a particular gesture and time, as well as a table with a number of gestures performed during the test, mean number of gestures performed in one second and time taken for each gesture.

## Summary

As presented in this paper, owing to the analysis of biomedical signals and Internet video camera images, it was possible to monitor movement activity of patients with PD. The results obtained in experiments show that it is possible to identify motor activity of a PD patients based on the analysis of the acceleration signals. The high effectiveness and scalability of the described approach makes it practically feasible for a 24-h monitoring of patients. They may also constitute a basis for designing algorithm of a higher level, for example, for the analysis of PD symptoms such as FOG or slowness of hand movement. In the future, the algorithms based on video image analysis should allow for the creation of more objective parameters of patients’ evaluation that will be based on the results of the proposed tests.
